# A mini-review on functional magnetic resonance imaging on brain structure of vascular cognitive impairment

**DOI:** 10.3389/fneur.2023.1249147

**Published:** 2023-09-21

**Authors:** Zeyi Wei, Jinhuan Yue, Xiaoling Li, Weiwei Zhao, Danna Cao, Ang Li, Guanhu Yang, Qinhong Zhang

**Affiliations:** ^1^Graduate School of Heilongjiang University of Chinese Medicine, Harbin, China; ^2^Shenzhen Frontiers in Chinese Medicine Research Co., Ltd., Shenzhen, China; ^3^Department of Acupuncture and Moxibustion, Vitality University, Hayward, CA, United States; ^4^Division of CT and MRI, First Affiliated Hospital of Heilongjiang University of Chinese Medicine, Harbin, China; ^5^MSD R&D (China) Co., Ltd., Beijing, China; ^6^Servier (Beijing) Pharmaceutical Research & Development CO. Ltd., Beijing, China; ^7^Department of Specialty Medicine, Ohio University, Athens, OH, United States; ^8^Department of Acupuncture and Moxibustion, Heilongjiang University of Chinese Medicine, Harbin, China

**Keywords:** vascular cognitive impairment, functional magnetic resonance imaging, structural magnetic resonance imaging, low-frequency amplitude, local consistency, functional connectivity

## Abstract

Vascular cognitive impairment (VCI) is the initial stage of vascular dementia (VaD). Early diagnosis and treatment of VCI are crucial to prevent the progression of VaD. In order to gain a better understanding of VCI, this study aimed to investigate the use of advanced imaging techniques such as structural magnetic resonance imaging (sMRI) and resting-state functional magnetic resonance imaging (rs-fMRI). These techniques allow researchers to observe the structural and functional changes in the brain that are associated with VCI. Functional magnetic resonance imaging (fMRI) and sMRI techniques have been widely used in studies focusing on gray matter, brain networks, and functional abnormalities during rest. By searching and summarizing recent literature, this study has provided valuable evidence on the use of advanced imaging techniques in understanding and treating VCI. The findings from this study can aid in the development of early intervention strategies for patients with VCI, potentially slowing down or even halting the progression of VCI to full-blown VaD.

## Introduction

Vascular cognitive impairment (VCI) is the second most common type of dementia (1, 2). It encompasses a spectrum of cognitive disorders, from mild VCI (mVCI) to vascular dementia (VaD) (3–5). Of those, mVCI refers to mild cognitive impairment caused by vascular factors, while VaD refers to more severe cognitive decline associated with extensive vascular injury. The classification of VCI into mVCI and VaD has been established by the vascular impairment of cognition classification consensus study (VICCCS) (6, 7). This categorization helps in better understanding and diagnosing VCI. Within the category of VaD, there are four subtypes, namely, post-stroke dementia, subcortical ischemic vascular dementia (SIVD), multi-infarct (cortical) dementia, and mixed dementia (8). Each subtype has its specific features and characteristics. VCI is a significant contributor to the overall burden of dementia, accounting for over 20–40% of dementia cases (9). This emphasizes the importance of recognizing and understanding VCI as a distinct entity. Traditionally, VaD has been differentiated from AD based on clinical manifestations, history of vascular disorders, and findings on neurological examination. However, recent evidence suggests that cerebral vascular diseases play a crucial role in the development and progression of AD and other neurodegenerative disorders (10). This highlights the overlap and interplay between vascular and neurodegenerative pathologies in cognitive impairment.

Functional magnetic resonance imaging (fMRI) is a valuable tool for studying VCI. It is a non-invasive and non-radiative imaging technique that provides high-resolution structural and functional images of the brain (11). Two commonly used methods in fMRI for detecting changes in VCI are structural magnetic resonance imaging (sMRI) and resting-state functional magnetic resonance imaging (rs-fMRI). Of these, sMRI is a non-invasive imaging technique used to study the brain’s structure and identify abnormalities. By examining the structural changes in organs, such as growth, atrophy, and deformation, sMRI can provide detailed insights into the morphology of the brain. Rs-fMRI is a technique that is used to detect spontaneous brain activity in individuals at rest, allowing researchers to explore brain function (12, 13). It is used to investigate the mapping of brain function, aiming to understand the activation of certain brain regions in response to specific brain activities. By studying the brain’s intrinsic activity, rs-fMRI can provide valuable information about various aspects of brain function without requiring the participants to perform specific tasks. In summary, sMRI primarily focuses on observing changes in brain structures, while rs-fMRI is used to investigate brain function. For example, sMRI can reveal if there are any changes in brain volume or shape associated with a particular condition. On the other hand, rs-fMRI can determine which brain regions are active during specific tasks or identify abnormal brain activity associated with certain diseases. These techniques allow researchers to examine both structural abnormalities and functional connectivity alterations in VCI.

### SMRI study of VCI

In the case of VCI, the diagnosis is performed using T_1_ weighted imaging, T_2_ weighted imaging, and fluid-attenuated inversion recovery, which are special imaging sequences used to measure the thickness, density, and volume of the cerebral cortex with high accuracy. This allows for the quantitative identification of lesions in the brain. The imaging features of VCI mainly involve cerebrovascular disease ischemic injury, which is predominantly found in the parietal cortex, hippocampus, and basal ganglia. These areas of the brain are particularly affected by VCI. Additionally, brain atrophy and hemorrhagic changes are also common findings in sMRI scans of individuals with VCI. It is important to note that the location and types of lesions can vary among individuals, leading to heterogeneity in the corresponding cognitive impairments experienced by patients with VCI (14).

In a study conducted by Zhao and colleagues, the researchers aimed to explore the value of sMRI and the APOE-ε4 genotype in the early diagnosis and progression of senile VCI without dementia (VCIND) (15). The results of the study revealed several significant findings. Patients with VCIND were found to have shorter education durations, a higher prevalence of hypertension, elevated levels of homocysteine, and lower cognitive abilities compared to the healthy control group. Furthermore, sMRI scans showed that patients with VCIND had increased white matter volume, white matter hyperintensity (a marker of small vessel disease in the brain), lacunar infarction (small areas of tissue damage due to lack of blood flow), and higher Fazekas scores (a scale used to quantify white matter changes). These findings suggest that individuals carrying the APOE-ε4 genotype, a genetic variant associated with increased risk for Alzheimer’s disease and vascular-related conditions, are more susceptible to developing VCIND. These findings highlight the potential of combining sMRI with APOE-ε4 genotype in the early diagnosis and monitoring of VCIND progression in elderly patients. This approach could facilitate timely intervention and management strategies to improve the outcomes of individuals with VCI.

In their study, Tan and colleagues aimed to investigate the structural imaging changes occurring in patients with SIVD-VCIND and SIVD-VaD, which are subtypes of SIVD (16). They found that gray matter atrophy was observed in multiple brain regions in both groups. Specifically, in the SIVD-VCIND group, it was detected in the bilateral orbitofrontal gyrus, right middle temporal gyrus, superior temporal gyrus, and anterior cuneiform lobe (16). This suggests that the patients with SIVD-VCIND showed degeneration in these areas of the brain. On the other hand, the SIVD-VaD group exhibited gray matter atrophy in the left cerebellum 6, cerebellum peduncle 1, bilateral thalamus, right anterior cuneiform lobe, and corpus callosum. These findings indicate that patients with SIVD-VaD experienced degenerative changes in these particular brain regions. Overall, these results suggest that both SIVD-VCIND and SIVD-VaD are associated with extensive and subtle structural changes in the gray matter of the brain. Such alterations may play a crucial role in the development and manifestation of subcortical VCI. The findings of this study provide valuable evidence that helps in explaining the underlying mechanisms of subcortical VCI (16). It highlights the importance of identifying and understanding the structural changes occurring in the brain, which can contribute to better diagnosis, management, and treatment strategies for patients with subcortical VCI.

### Rs-fMRI study of VCI

In the field of VCI research, rs-fMRI has been applied to investigate different aspects of brain activity, including the amplitude of low-frequency fluctuations (ALFF), regional homogeneity (ReHo), and functional connectivity (FC) (17, 18).

In a study conducted by Zhang and colleagues, the changes in ALFF/functional ALFF (fALFF) were examined in individuals with amnesiac-mild cognitive impairment (aMCI) and vascular mild cognitive impairment (vMCI) compared to healthy controls (HCs) (19). The results showed that the ALFF/fALFF values were significantly decreased in patients with aMCI when compared to HCs. Interestingly, relative to HCs, individuals with aMCI exhibited an increase in ALFF/fALFF in several brain regions, including the bilateral parahippocampal gyrus/hippocampus, right amygdala, right anterior cerebellar lobe, left middle temporal gyrus, left inferior temporal gyrus, left inferior temporal gyrus, and left cerebrum limbic lobe uncus. However, there was a decrease in ALFF/fALFF in the bilateral precuneus, bilateral cuneus, and bilateral posterior cingulate regions in patients with aMCI. Similarly, when patients with vMCI were compared to HCs, there was a decrease in ALFF/fALFF values observed in the bilateral cuneus, left precuneus, left PC, and right cingulate gyrus. These findings indicate that there are distinct patterns of altered brain activity in both aMCI and vMCI patients compared to HCs. The results of this study suggest that the examination of ALFF/fALFF values can serve as a reliable tool to differentiate between aMCI and vMCI and provide insights into the underlying pathophysiology of these disorders.

In their study, Diciotti and colleagues utilized rs-fMRI to analyze the whole brain and evaluate its relationship with cognitive test scores (20). They found that the ReHo values, which indicate the local synchronization of neural activity, were significantly negatively correlated with Montreal Cognitive Assessment (MoCA) scores. This suggests that patients with lower MoCA scores, indicating more severe cognitive impairment, had higher ReHo values. Interestingly, the researchers also discovered that the ReHo values of the left posterior cerebellum (specifically Crus 1) were higher in patients with severe cognitive impairment. This suggests that this particular region may be associated with cognitive deficits in subcortical VCI. In addition to the negative correlation with MoCA scores, the study also found a significant positive correlation between ReHo values and Stroop scores. The Stroop test measures an individual’s ability to inhibit automatic responses, which is an aspect of executive function. Patients with poorer executive function had higher ReHo values in the bilateral middle cingulate cortices. This suggests that increased activity in these regions may serve as an adaptive or compensatory mechanism for specific cognitive deficits.

The study by Chang and colleagues aimed to explore the FC and neurobehavioral dysfunction within the brain network in patients with VCIND (21). They also investigated the association between cognitive scores, FC, and physical activity (PA).

The results of their study showed that FC in the caudal anterior cingulate cortex (ACC) seeds, which anchored the brain network, was positively correlated with higher physical activity levels, as indicated by more average steps and mean distance. Better Instrumental Activities of Daily Living (IADL) scores, which reflect an individual’s ability to live independently, were also associated with higher FC in these brain regions. On the other hand, FC was negatively correlated with longer walk test B time to completion (s), suggesting that patients with slower completion time had lower FC in the brain network anchored by the caudal ACC seeds. This implies that disruptions in the integrity of this network may contribute to impairments in physical performance. Furthermore, the study found that FC in the brain network anchored by the putamen, a subcortical structure involved in motor control, was positively associated with higher average calorie consumption and better IADL scores. This suggests that the functionality of this specific brain network may influence not only physical abilities but also aspects of daily living and energy expenditure. Overall, these findings highlight the importance of understanding the relationship between brain connectivity, cognitive function, and physical activity in patients with VCI. The study suggests that alterations in brain networks may contribute to cognitive deficits and that physical activity could potentially impact neurobehavioral functions through the injured anterior cingulate cortex and striatal network.

### Limitations

There are still several limitations in the study of VCI using multimodal MRI. First, in addition to typical cerebrovascular diseases, such as strokes or small vessel disease, it is important to investigate potential brain structural differences in cognitive impairment caused by VCI compared to other causes. For example, neurodegenerative conditions such as Alzheimer’s disease or Lewy body dementia might share similar cognitive symptoms with VCI, making it crucial to examine if there are specific structural variations that differentiate VCI from these disorders. Second, the influence of patients’ pathological and physiological state on cerebral blood flow changes during fMRI examination needs further clarification. Factors such as medication usage, comorbidities, or even individual variations in systemic blood pressure can potentially impact cerebral blood flow measurements during fMRI. Investigating these factors will help determine the extent to which the observed changes in cerebral blood flow are directly related to VCI pathology. Third, exploring potential differences in brain structure or function between VCI and other diseases is essential for both diagnostic and treatment purposes. By understanding the unique characteristics of VCI, clinicians can potentially develop more targeted interventions. Additionally, this can aid in distinguishing VCI from other conditions with similar cognitive symptoms, leading to more accurate diagnoses and tailored treatment plans. In summary, several limitations in the study of VCI using MRI need to be addressed. These include understanding brain structural differences compared to other causes of cognitive impairment, clarifying the impact of patients’ pathological and physiological state on cerebral blood flow changes during fMRI, and exploring differences in brain structure or function between VCI and other diseases.

## Summary

In conclusion, multimodal MRI plays a crucial role in identifying alterations in both the structure and function of the brain even before any morphological changes occur in individuals with VCI (22). This highlights the significance of using multimodal MRI as a reliable tool for early diagnosis, intervention, treatment, and prognosis of VCI. It is imperative for future studies to concentrate on conducting comparative research that explores both the morphological and functional aspects of multimodal MRI imaging for VCI. Additionally, it would be valuable to perform comprehensive analyses of brain changes using alternative imaging techniques in order to corroborate and complement the findings obtained through multimodal MRI.

Further research should be dedicated to exploring the potential application of multimodal MRI in the evaluation and treatment of VCI. It is possible to combine multimodal MRI techniques to conduct large-scale, multi-center longitudinal studies. In addition to studying brain structure and brain functional activities, further research can be done on VCI brain metabolism and cerebral blood perfusion. Moreover, the application and development of neural networks, machine learning, and deep learning in image processing can enhance the accuracy of diagnosis, extracting more image information from difficult-to-obtain data, and providing stronger support for accurate diagnosis of VCI. These ground-breaking findings provide compelling evidence that enables accurate diagnosis of VCI, leading to improved patient care and outcomes.

## Author contributions

Concept and design: ZW, JY, XL, and QZ. Data curation: ZW, XL, and QZ. Formal analysis: not reported. Funding acquisition: XL and DC. Investigation: QZ, AL, and GY. Methodology: WZ and AL. Project administration: QZ, AL, and GY. Resources: ZW, JY, DC, and XL. Software: not reported. Supervision: QZ, AL, and GY. Validation: All authors. Visualization: All authors.

## Funding

The author(s) declare financial support was received for the research, authorship, and/or publication of this article. This study was partly funded by the National Foundation of Natural Science of China (82074537 and 81373714); and the Joint Guidance Project of Natural Science Foundation of Heilongjiang Province (LH2020H103 and LH2021H101). The supporters had no roles in any part of this study.

## Conflict of interest

WZ was employed by the MSD R&D (China) Co., Ltd., AL was employed by Servier (Beijing) Pharmaceutical Research & Development CO. Ltd. JY and QZ were employed by the Shenzhen Frontiers in Chinese Medicine Research Co., Ltd.

The remaining authors declare that the research was conducted in the absence of any commercial or financial relationships that could be construed as a potential conflict of interest.

## Publisher’s note

All claims expressed in this article are solely those of the authors and do not necessarily represent those of their affiliated organizations, or those of the publisher, the editors and the reviewers. Any product that may be evaluated in this article, or claim that may be made by its manufacturer, is not guaranteed or endorsed by the publisher.
